# Precision in Prosthodontic Preparation: An In Vitro Study on the Digital Evaluation of Total Occlusal Convergence Angles Using Varying Magnification Loupes

**DOI:** 10.7759/cureus.99523

**Published:** 2025-12-18

**Authors:** Sneha Moturi, Tapan K Patro, Angurbala Dhal, Lokanath Garhnayak, Ullash Kumar

**Affiliations:** 1 Department of Prosthodontics and Crown & Bridge, SCB Dental College and Hospital, Cuttack, IND

**Keywords:** crown and bridge prosthesis, crown preparation, fixed prosthodontics, galilean loupes, keplerian loupes, magnifying loupes, precise crown preparation, tooth preparation techniques, total occlusal convergence angle, visual acuity outcome

## Abstract

Background

Precise control of the total occlusal convergence (TOC) angle during tooth preparation is essential for the long-term success of fixed dental restorations. While magnifying loupes combined with proper illumination enhance visual acuity, their influence on the precision of the TOC angle during preparation remains insufficiently studied.

Objective

This study aimed to evaluate the impact of magnification loupes on TOC angle accuracy. Specifically, it compared mean TOC values in two planes for tooth preparations performed without magnification, with 3.2× Galilean loupes, and with 6× Keplerian loupes.

Materials and methods

Ninety right mandibular first molar typodont teeth were randomly assigned to three groups (n=30 each): control (no magnification), 3.2× Galilean, and 6× Keplerian. A single trained operator, who was a postgraduate student with formal training in tooth preparation techniques but no prior experience of using magnification loupes, performed standardized full-coverage crown preparations. Each specimen was mounted on a custom die base to ensure consistent positioning. Digital images were captured in buccolingual and mesiodistal planes, and TOC angles were measured using computer screen protractor software, referencing the gingival 2 mm of the axial wall following a study by Yoon et al. A blinded evaluator recorded all measurements. Statistical analysis was conducted using one-way analysis of variance (ANOVA) and Tukey’s post hoc test (α=0.05).

Results

Both magnification groups demonstrated significantly lower TOC angles compared to the control group in both planes (P=0.001). The 6× Keplerian group achieved significantly lower TOC angles than the 3.2× Galilean group in the mesiodistal plane, though no significant difference was observed in the buccolingual plane.

Conclusion

The use of magnification loupes enhances the precision of TOC angle assessment during tooth preparation. Higher levels of magnification, especially with 6× Keplerian loupes, provide superior control in the mesiodistal dimension, thereby contributing to improved clinical outcomes in prosthodontic procedures.

## Introduction

Tooth preparation involves the removal of enamel, dentin, and cementum to prepare a tooth for the reception of a restoration [1]. Taper refers to the inclination of one wall of a preparation to the long axis of a tooth, whereas total occlusal convergence (TOC) refers to the angle formed between two opposing walls of the preparation in a given plane. In principle, the closer these walls are to parallel, the greater the retention achieved. As TOC increases, retention and resistance diminish. Parallel axial walls provide maximum stability, while highly convergent walls offer the least. It was previously established that the retention decreases in a hyperbolic manner as TOC rises, with retention reduced by half once TOC exceeds five degrees [2]. Consequently, maintaining a controlled taper and an acceptable TOC is fundamental to achieving mechanical stability, ensuring accurate seating of the restoration, and promoting long-term success in prosthodontic treatment.

A systematic review, evaluating different parameters in clinical crown preparation, identified TOC as the most critical factor influencing the longevity of fixed dental restorations. While early dental literature recommended an ideal TOC angle of 2 to 5 degrees, achieving such minimal taper is often impractical in clinical settings due to factors such as limited operator visibility, variations in tooth morphology, patient movement, and the need to preserve tooth structure while ensuring proper seating of the restoration. These challenges have led to a shift toward more clinically attainable values. Consequently, contemporary evidence supports a realistic range of 10 to 22 degrees, which balances mechanical stability with clinical feasibility [3,4].

Nevertheless, studies consistently report that both dental students and practicing clinicians often fail to achieve TOC angles within this recommended range [5-8]. Excessive tapering of preparations can occur due to restricted visual and mechanical access, improper instrumentation, anatomical variations, and operator-dependent factors such as training, visual acuity, hand-eye coordination, tactile sensitivity, and understanding of tooth preparation principles [9].

 Modern optical aids like binocular loupes (Galilean and Keplerian systems), when used with suitable lighting, have demonstrated advantages across different dental disciplines. These devices enhance the visual performance of the operator by providing three-dimensional object visualization, a wider field of view, and improved depth perception [10-14]. Compared to basic loupes, binocular loupes are lightweight and have been reported by Wajngarten et al. to offer distinct advantages. However, their use is recommended only after appropriate professional training [15].

Christensen [16] previously suggested that low-power magnification using 2.5× is suitable for procedures such as cavity preparation, while magnification of 4× or higher is preferable for tooth preparation or seating of crowns on lower anterior teeth. Additionally, Leknius and Geissberger reported that dental students who used magnification during prosthodontic procedures made fewer errors when compared to students who did not [17]. Eichenberger et al. documented improvements in visual performance using various magnification devices under proper illumination, noting enhancements in visual acuity ranging from 25 to 38 times with the use of such magnification devices [18]. Other studies have demonstrated that Keplerian loupes enhance the operator’s ability to detect fine details and improve overall precision, offering superior visual performance compared with Galilean loupes [19]. James and Gilmour [20] highlighted the importance of using magnification devices along with illumination, reporting significant improvements in visual acuity when a fiberoptic light source was used. Similarly, in 2001, researchers concluded that real-time magnification significantly enhanced the undergraduate students’ understanding of taper during preclinical teaching [21].

Recent studies continue to highlight the critical role of magnification in prosthodontics, demonstrating its value in enhancing visual acuity, improving preparation accuracy, and supporting more predictable clinical outcomes. Braga et al. observed higher pass rates among students using loupes in simulation labs [22]. Atlas et al. compared CAD-CAM crown margins fabricated with loupes versus microscopes and found significantly reduced marginal gaps with higher magnification [23]. Nóbrega et al. evaluated finish line quality and found slight improvements with low-power magnification, though results were not statistically significant [24]. In contrast, Murbay et al. assessed TOC angles in preparations performed by predoctoral students using 2.5× magnification and found no significant differences compared to those without magnification; however, these procedures were conducted under inadequate illumination, which may have influenced the results [25].

Although magnification has been extensively investigated in prosthodontic procedures, few studies have specifically examined its effect on TOC angles. Importantly, no research to date has compared the influence of Galilean and Keplerian optical systems on TOC angles under adequate illumination. The present study, therefore, aimed to quantitatively assess TOC angles, expressed in degrees, both with and without the use of magnifying loupes. The null hypothesis proposed that no significant differences would be found in TOC angles between preparations performed with or without magnification, nor between those completed using 3.2× Galilean and 6× Keplerian loupes.

## Materials and methods

This in vitro study was approved by the Institutional Ethics Committee (IEC/SCBDCH/225/2023) on 09/08/23. A sample size of 81 was calculated based on an alpha error of 5%, 80% power, and a large effect size [26]. Considering an attrition of 10%, a final sample size of 90 was calculated. These samples were equally divided into three groups: control group (n=30), 3.2× Galilean group (n=30), and 6× Keplerian group (n=30). Typodont teeth (Nissin Dental Products, Kyoto, Japan) were mounted in artificial jaws by the same manufacturer and prepared for full veneer metal-ceramic restoration by a trained operator using standardized handpieces and diamond rotary burs. The operator was a postgraduate student in the Department of Prosthodontics and Crown & Bridge, Cuttack, India, with formal training in tooth preparation techniques but no prior experience of using magnification loupes. To ensure adequate adaptation, five additional samples were prepared using 3.2× magnification and five using 6× magnification before commencing the study. These preliminary preparations were performed solely for calibration and were not included in the final evaluation.

In the control group, tooth preparations were performed without magnifying loupes, using only the dental chair light at an intensity of 25,000 LUX. In the magnification groups, preparations were carried out with 3.2× Galilean loupes (Admetec, Israel) and 6× Keplerian loupes (Admetec, Israel), each fitted with Orchid headlights (Admetec, Israel) that provided 85,000 LUX illumination. The difference in illumination reflects the inherent design of loupe systems, which include stronger integrated lighting to enhance visibility [19]. Tooth reduction followed anatomical contours and was made using standardized protocols for full veneer metal ceramic restorations. They were made using NSK® standard head LED airotor (Tokyo, Japan) with Shofu® Regular grit and fine grit tapered round burs (#102R, #SF102R). First, occlusal depth orientation grooves were placed using a tapered, small round end bur. Occlusal reduction was performed using the #102R coarse-grained diamond drill for a 1-1.5 mm reduction. Axial walls were prepared to a reduction of 1.2-1.5 mm using the #102R diamond drill, and, finally, a fine-grit bur was used to finish the prepared typodont tooth surface. Tooth reductions respected the tooth’s anatomy, and due to the absence of pre-existing cavities and restorations, a standardized preparation technique was ensured. While preparing the typodont tooth samples, the operators’ visual axis aligned with the light source. While seated in the dental chair, the operator maintained a head position of 25 degrees forward while sitting upright. Their feet rested flat on the floor, with their knees positioned below, enabling direct visibility and binocular vision to prepare all samples [1]. A minimum of 4 mm abutment height was maintained in all samples to ensure adequate retention and resistance [4]. The preparations were performed on two typodont samples, per group per day, and were repeated one or two days later under faculty supervision, ensuring consistency across groups.

**Figure 1:**
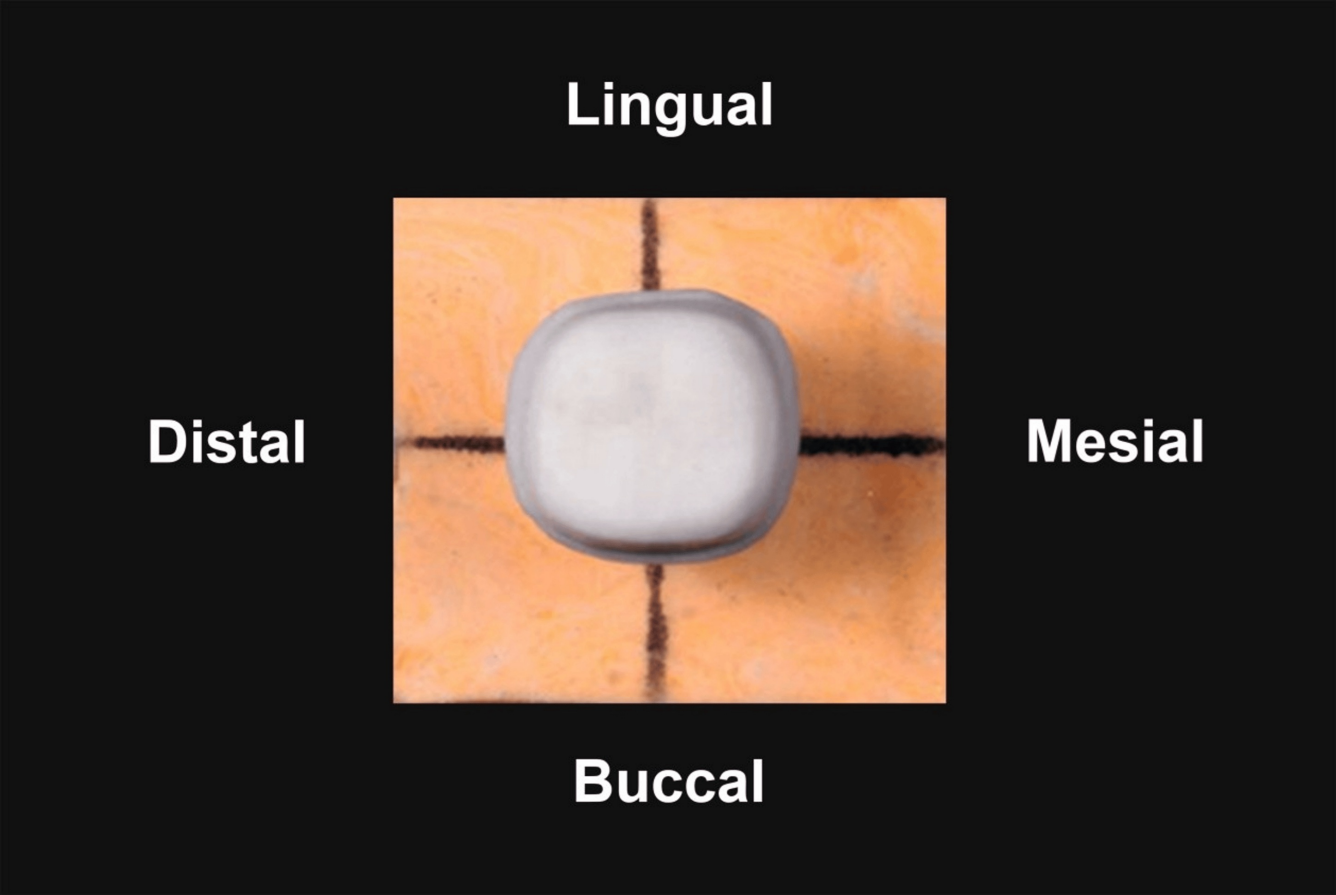
Custom die base with prepared typodont tooth Typodont tooth marked on buccal, mesial, lingual and distal surfaces for consistent repositioning.

Custom die bases were fabricated using addition silicone (Perfit Elastomeric impression material, Type 0 putty, normal set, Lot 2411142, Shandong, China) to provide stable and reproducible positioning. Each tooth was aligned with its long axis perpendicular to the base using a dental surveyor and was held firmly until the base material had set. External landmarks were marked on buccal, mesial, lingual, and distal surfaces of the bases to facilitate rotation and re-alignment, enabling image capture in both buccolingual and mesiodistal planes (Figure 1).

**Figure 2:**
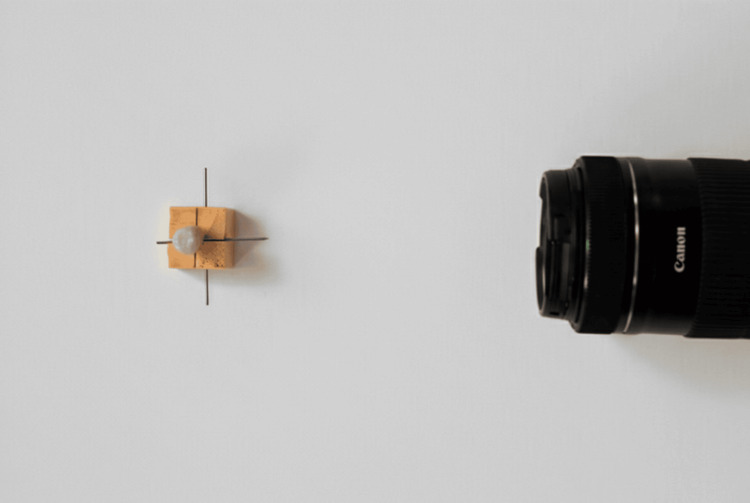
Positioning camera lens 30 cm from custom die base Images captured in buccolingual and mesiodistal orientations for each typodont sample.

These reference points ensured consistent positioning across all specimens. Digital images were captured using a Canon EOS D700 camera equipped with a Canon EF 100 mm f/2.8 macro lens, mounted on a mini tripod at a fixed distance of 30 cm from the custom die base (Figure 2).

**Figure 3:**
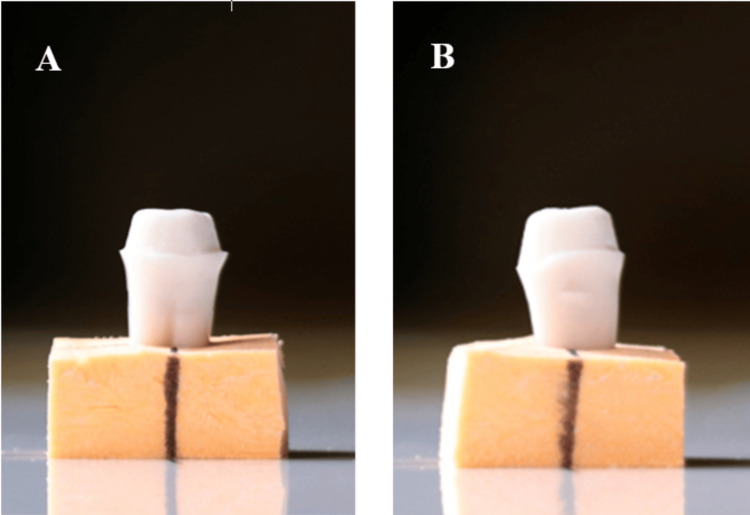
Prepared typodont tooth A: buccolingual plane; B: mesiodistal plane.

A dark background was used to enhance contrast between the typodont tooth and the background, improving visualization of axial walls on the computer screen. For each sample, two images were taken, one in the buccolingual plane (Figure 3A) and one in the mesiodistal plane (Figure 3B).

**Figure 4:**
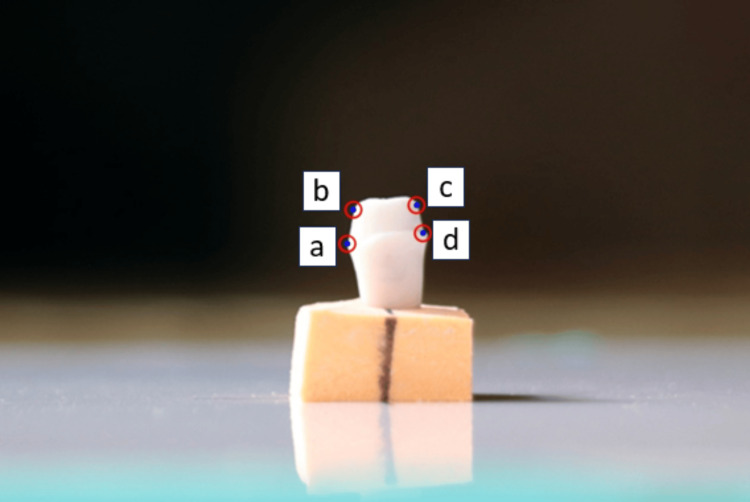
Location of points for measurement of TOC Points (a, b) were located on the left axial wall and Points (c, d) were located on the right axial wall. TOC, total occlusal convergence.

Each image was magnified 15 times to clearly see the gingival 2 mm of each axial wall that was critical for retention and resistance form, as conducted by Yoon et al. [9]. On each image, two outermost points, point a and point b, were identified on the left axial wall, and two points, point c and point d, were located on the right axial wall (Figure 4).

**Figure 5:**
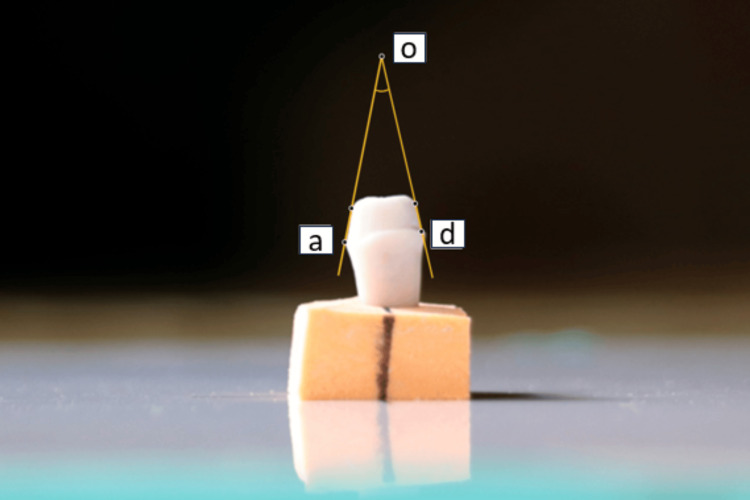
Intersection of lines to meet at Point o Line extended from Points (a, b) and (c, d) to intersect at Point o.

Lines passing through points (a, b) and (c, d) were extended until they intersected at point o (Figure 5).

**Figure 6:**
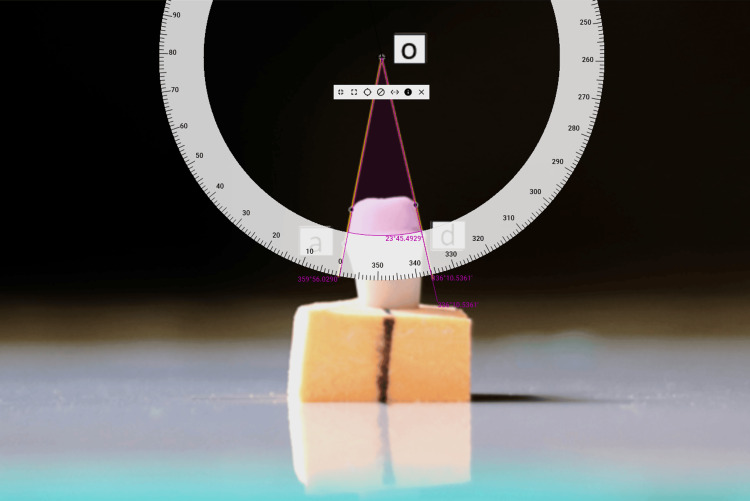
Measurement of TOC angle Angles between lines aod were measured using computer screen protractor software. TOC, total occlusal convergence.

The TOC angle was defined as the angle formed at o between lines (aod). Measurements were performed using computer screen protractor software (Pissa Ruler, v1.0.40, IO Stream, Microsoft Store) by an investigator blinded to group allocation (Figure 6).

Intra-examiner reliability was assessed and found to be moderate to good across groups: The intraclass correlation coefficient (ICC) values for the control group (ICC=0.93; P=0.003), 3.2× Galilean group (ICC=0.64; P=0.05), and 6× Keplerian group (0.88; P=0.006) were statistically significant.

Data analysis was performed using SPSS for Windows (version 26.0, IBM Corp., Armonk, NY). Continuous variables were compared using one-way ANOVA followed by post hoc Tukey testing. Categorical data were analyzed using the chi-square test. Results were presented in tables and graphs. The level of statistical significance was set at P <0.05.

## Results

**Figure 7:**
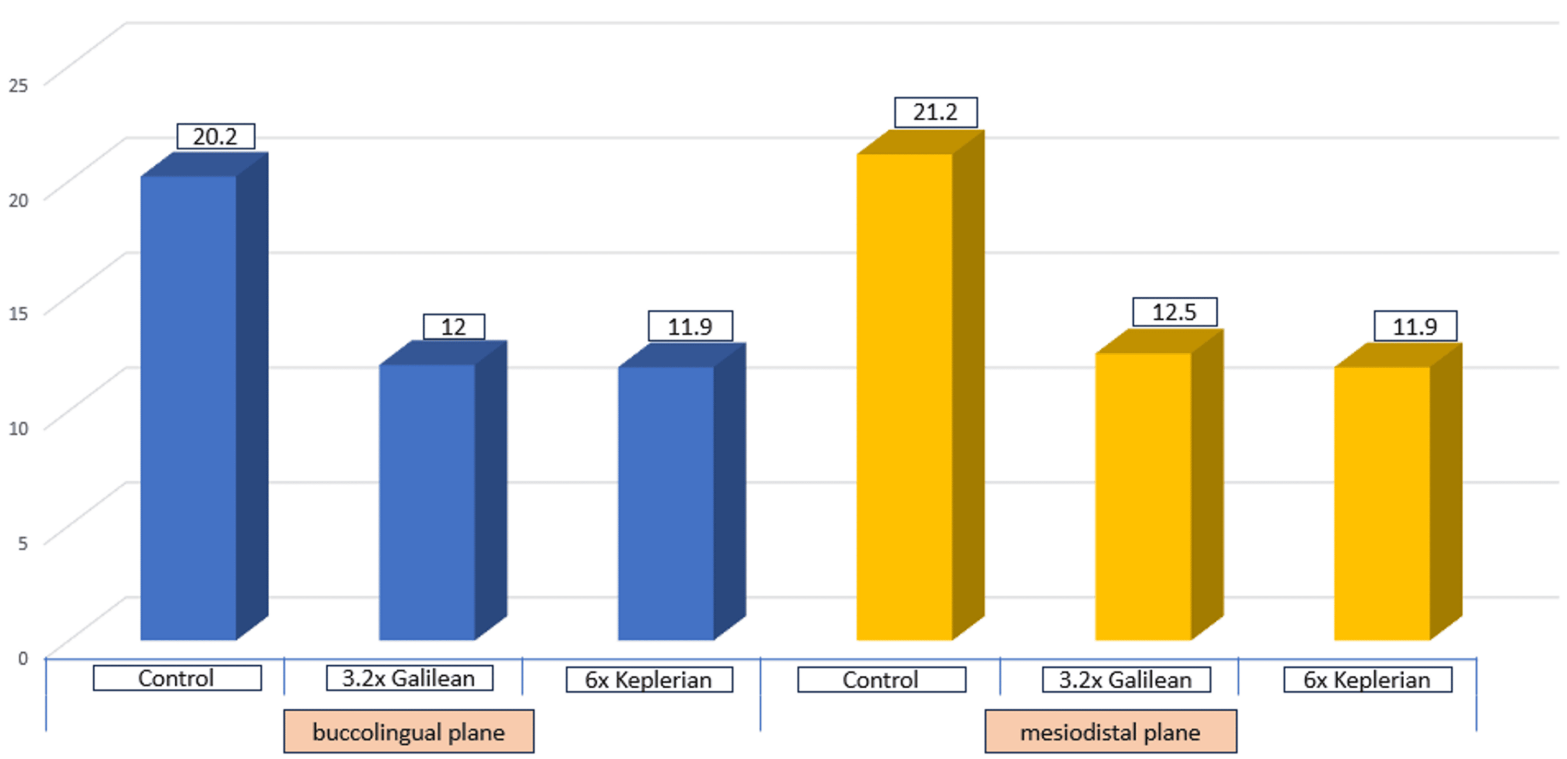
Depiction of mean TOC angles in buccolingual and mesiodistal planes among three groups TOC, total occlusal convergence.

In the control group, TOC angles in the buccolingual plane ranged from 18.8 degrees to 22.4 degrees, with a mean of 20.2±1.1 degrees. In the mesiodistal plane, values ranged from 19.4 degrees to 22.8 degrees, with a mean of 21.2±1.0 degrees. In the 3.2× Galilean group, buccolingual TOC angles ranged from 11.1 degrees to 12.9 degrees, with a mean of 12.0±0.5 degrees, while mesiodistal values ranged from 11.6 degrees to 14.3 degrees, with a mean of 12.5±1.4 degrees. For the 6× Keplerian group, buccolingual values ranged from 11.3 degrees to 12.8 degrees, with a mean of 11.9±0.4 degrees, and mesiodistal values ranged from 11.1 degrees to 13.4 degrees, with a mean of 11.9±0.5 degrees (Table 1; Figure 7).

**Table 1:** Comparison of mean total angle of convergence with reference to buccolingual plane and mesiodistal plane SD, standard deviation; ANOVA, analysis of variance. *Statistically significant using one-way ANOVA.

Plane	Control		3.2× Galilean		6× Keplerian		F	P-value
	Mean	SD	Mean	SD	Mean	SD		
Buccolingual plane	20.2	1.1	12	0.5	11.9	0.4	1292.3	0.001*
Mesiodistal plane	21.2	1.0	12.5	1.4	11.9	0.5	754.5	0.001*

Statistical analysis confirmed significant differences in mean TOC angles among the groups in both buccolingual and mesiodistal planes (P=0.001; Table 1). Post hoc Tukey test revealed significant differences between the control group and both magnification groups. Additionally, in the mesiodistal plane, the 6× Keplerian group demonstrated significantly lower TOC angles compared with the 3.2× Galilean group (Table 2).

**Table 2:** Multiple comparison tests among the groups MD, mean difference. *Statistically significant using the Tukey test.

Plane	Group 1	Group 2	MD	P-value
Buccolingual plane	Control	3.2× Galilean	8.2	0.001*
	Control	6× Keplerian	8.3	0.001*
	3.2× Galilean	6× Keplerian	0.09	0.86
Mesiodistal plane	Control	3.2× Galilean	8.64	0.001*
	Control	6× Keplerian	9.2	0.001*
	3.2× Galilean	6× Keplerian	0.61	0.001*

## Discussion

There is limited evidence regarding the impact of different magnifying loupes on the quality of full veneer crown preparations. This in vitro study quantitatively evaluated the TOC angles of typodont teeth prepared for full veneer crown restorations by a postgraduate student using two types of magnifying loupes: 3.2× Galilean and 6× Keplerian. The TOC angle was selected as the primary parameter for assessing the preparation quality, as it is widely regarded as the most critical factor influencing retention and resistance of full veneer restorations, as highlighted in the systematic review by Tiu et al. [3].

The mandibular right first molar was chosen to minimize confounding variables, providing direct vision and ease of access during tooth preparation [25]. TOC angles were measured using the digital quantitative method described by Yoon et al. [9]. The null hypothesis was rejected, as statistically significant differences were observed in TOC angles between preparations performed with 3.2× and 6× magnifying loupes compared to the control group. Furthermore, significant differences were noted in the mesiodistal plane when using 6× Keplerian loupes.

Ideal TOC angles reported in early dental literature ranged from 2 to 5.5 degrees. Later, Goodacre et al. recommended a more clinically achievable range of 10 to 20 degrees, along with a minimum occlusocervical dimension of 4 mm for molars [4]. More recent systematic reviews have confirmed that the TOC angle remains the most important factor for retention and resistance, with a realistic taper range of 10 to 22 degrees [3].

Despite these guidelines, multiple studies have shown that both students and clinicians frequently exceed the recommended taper, leading to compromised retention [5-8]. For example, Winklemeyer et al. found that only 4.3% of zirconia-based preparations met clinical requirements, with mean TOC angles for molars of 23.2±10.1 degrees [5]. Similarly, Sadid-Zadeh et al. found that 95% first molar preparations submitted to laboratories exceeded 20 degrees [6]. General dental practitioners preparing zirconia crowns demonstrated mean tapers of 26.7 degrees for molars [7]. Even under preclinical conditions, dental students often produced excessive taper, with third-year students averaging nearly 20 degrees and fourth-year students averaging 14 to 16 degrees [8].

Excessive tapering may be mitigated by magnification devices, which enhance stereopsis, field of view, and depth perception, thereby improving precision [10]. Galilean loupes utilize concave ocular lenses paired with convex objective lenses, whereas Keplerian loupes incorporate convex lenses and prisms. Compared to Galilean systems, Keplerian loupes offer higher magnification, greater depth of field, wider field of view, and longer focal length [12-14].

Murbay et al. digitally evaluated the use of 2.5× magnifying loupes in complete coverage crown preparations, and found that nearly half of the students failed to achieve TOC angles within the 10 to 22 degrees range, despite using low-power magnification. However, their study involved predoctoral students working under low illumination, which may explain the discrepancy with the present findings [25].

In the control group, the mean TOC angle in the buccolingual plane was 20.2±1.1 degrees, and 21.2±1.03 degrees in the mesiodistal plane, both exceeding the recommended TOC range. In contrast, the 3.2× Galilean group achieved mean TOC angles of 12±0.47 degrees (buccolingual plane) and 12.5±1.37 degrees (mesiodistal plane), while the 6× Keplerian loupes group achieved mean TOC angles of 11.9±0.41 degrees (buccolingual plane) and 11.9±0.49 degrees (mesiodistal plane). Statistically significant differences in TOC angles were observed between both magnification groups (3.2× Galilean and 6× Keplerian) and the control group. Additionally, significant differences were found in the mesiodistal plane between the 6× Keplerian and 3.2× Galilean groups, although no such difference was observed in the buccolingual plane based on a post hoc Tukey test.

These findings suggest that magnification enhances the operator’s ability to achieve clinically acceptable TOC angles, particularly in the mesiodistal plane, where higher magnification (6× Keplerian) provided superior precision. Overall, magnifying loupes facilitated a more uniform reduction in the buccolingual plane compared to the mesiodistal plane.

This study has several limitations. First, only mandibular molars were evaluated; future research should include teeth from all quadrants to enhance the generalizability of the findings. Second, the in vitro design may not fully replicate intraoral conditions, where patient movement, saliva, and restricted access can influence preparation outcomes. Third, the high cost of magnification equipment may limit its widespread use in routine clinical practice. Fourth, illumination levels were not standardized across groups. The magnification groups operated under higher lighting, which may have affected operator performance and preparation accuracy. Finally, the use of a single operator introduces potential bias, as individual techniques and skills may influence preparation outcomes. 

Clinical implication

Precise control of the TOC angle during tooth preparation is essential for adequate retention and the long-term success of fixed dental restorations. The findings of this study demonstrate that the use of magnifying loupes, particularly higher-magnification Keplerian loupes, significantly enhances the precision of the TOC angle compared with direct vision without magnification.

## Conclusions

Within the limitations of this in vitro study, the findings indicate that the use of magnifying loupes is associated with enhanced precision of TOC angles during tooth preparation. Preparations performed without magnification demonstrated the lowest accuracy, while increasing levels of magnification were linked to progressively improved outcomes. Notably, higher magnification with 6× Keplerian loupes yielded superior precision in the mesiodistal plane compared to 3.2× Galilean loupes.

However, these results should be interpreted cautiously, as illumination differences between groups and the use of a single operator may have influenced outcomes. The evidence presented supports the potential of magnification to improve preparation accuracy under controlled laboratory conditions, but confirmation through in vivo studies is necessary before clinical recommendations can be made.
